# Cost-effectiveness of health promotion targeting physical activity and healthy eating in mental health care

**DOI:** 10.1186/1471-2458-14-856

**Published:** 2014-08-18

**Authors:** Nick Verhaeghe, Delphine De Smedt, Jan De Maeseneer, Lea Maes, Cornelis Van Heeringen, Lieven Annemans

**Affiliations:** Department of Public Health, Ghent University, De Pintelaan, 185 9000 Ghent, Belgium; Department of Medical Sociology, Vrije Universiteit Brussel, Laarbeeklaan, 103 1090 Jette, Belgium; Department of Family Medicine and Primary Health Care, Ghent University, De Pintelaan, 185 9000 Ghent, Belgium; Department of Psychiatry and Medical Psychology, Ghent University, De Pintelaan, 185 9000 Ghent, Belgium

**Keywords:** Mental disorders, Cost-effectiveness, Health promotion, Physical activity, Healthy eating

## Abstract

**Background:**

There is a higher prevalence of obesity in individuals with mental disorders compared to the general population. The results of several studies suggested that weight reduction in this population is possible following psycho-educational and/or behavioural weight management interventions. Evidence of the effectiveness alone is however inadequate for policy making. The aim of the current study was to evaluate the cost-effectiveness of a health promotion intervention targeting physical activity and healthy eating in individuals with mental disorders.

**Methods:**

A Markov decision-analytic model using a public payer perspective was applied, projecting the one-year results of a 10-week intervention over a time horizon of 20 years, assuming a repeated yearly implementation of the programme. Scenario analysis was applied evaluating the effects on the results of alternative modelling assumptions. One-way sensitivity analysis was performed to assess the effects on the results of varying key input parameters.

**Results:**

An incremental cost-effectiveness ratio of 27,096€/quality-adjusted life years (QALY) in men, and 40,139€/QALY in women was found in the base case. Scenario analysis assuming an increase in health-related quality of life as a result of the body mass index decrease resulted in much better cost-effectiveness in both men (3,357€/QALY) and women (3,766€/QALY). The uncertainty associated with the intervention effect had the greatest impact on the model.

**Conclusions:**

As far as is known to the authors, this is the first health economic evaluation of a health promotion intervention targeting physical activity and healthy eating in individuals with mental disorders. Such research is important as it provides payers and governments with better insights how to spend the available resources in the most efficient way. Further research examining the cost-effectiveness of health promotion targeting physical activity and healthy eating in individuals with mental disorders is required.

## Background

The prevalence of overweight (Body Mass Index 25-29.9 kg/m^2^) and obesity (Body Mass Index ≥30 kg/m^2^) has increased in the last three decades and has become a serious global public health concern [1]. Obesity is a risk factor for the development of important non-communicable diseases including type 2 diabetes, coronary heart disease (CHD), stroke, and certain cancers such as colon cancer and breast cancer [2] leading to considerable healthcare expenditures [3]. Obesity is also associated with reduced health-related quality of life (HRQOL) [4] and with reduced life expectancy [5]. There is conclusive evidence that the burden of weight gain is even higher in individuals with mental disorders (MD) than in the general population [6, 7]. Important factors contributing to the high prevalence of overweight and obesity in people with MD are side effects of especially second generation antipsychotic drugs [8], a lack of regular physical activity (PA) and unhealthy eating behaviour [9].

Hence, health promotion interventions targeting PA and healthy eating should be integrated into the daily care of individuals with MD. The results of previous research suggested that weight loss following behavioural and/or psycho-educational programmes in MD patients is possible [10, 11]. Evidence on the effectiveness alone of such interventions is yet insufficient for policy making. Healthcare budgets are limited, hence policy makers are facing the problem how to set priorities in the allocation of healthcare resources to medical or public health interventions. Knowledge on this can be obtained by performing health economic evaluations of weight reduction programmes. The evidence derived from such research can assist regulatory bodies and health insurers establishing priorities within cost-constrained healthcare budgets. In the general population, health economic evaluations of health promotion programmes targeting PA [12] and healthy eating [13] yield mixed evidence on the cost-effectiveness of such interventions. In mental health care, such trials are lacking [14].

The aim of the current study was to evaluate the cost-effectiveness of a health promotion programme targeting PA and healthy eating in individuals with MD living in sheltered housing in the Flanders region in Belgium. Details on the design and results of the effectiveness study are described elsewhere [15]. In brief, the study design consisted of a cluster preference randomised controlled trial and was conducted in sheltered housing organisations (SHOs) in the Flanders region (Belgium). The intervention group included 16 SHOs (n = 201 individuals), while the control group included eight SHOs (n = 83 individuals). The health promotion intervention was based on an existing programme aimed at the general population developed by the Flemish Institute of Health Promotion and Disease Prevention [16]. As the target population of our study consisted of individuals with MD, some adjustments to the programme were made. The study consisted of a 10-week group-based and individually-based health promotion intervention, followed by a 6-month follow-up period. Individuals in the intervention group received the 10-week programme on top of their usual treatment, while those in the control group only received treatment as usual. The intervention was delivered by one or more mental health nurses working in the intervention SHOs. All participants in the intervention group received the same information in the same format comprising: (i) ten psycho-educational and behavioural group-based sessions in a 10-week period, (ii) group-based exercise in the same 10-week period (weekly 30’ supervised walking sessions), and (iii) individual support from the mental health nurses during the 10-week intervention. Data were collected at baseline, at ten weeks (end of the intervention) and after a six-month follow-up period.

## Methods

### Decision-analytic model

An age- and gender-dependent Markov decision-analytic model applying a public payer perspective was used to predict health outcomes and costs for the intervention and control arm. Health outcomes were expressed as quality-adjusted life years (QALYs) and calculated by multiplying the utility level (a HRQOL weight) for a given disease status with the number of years an individual is living with the disease. A utility of 1 equals perfect health, while 0 stands for death. In the cost dimension, both the programme costs and the disease costs were accounted for. Dividing the difference in costs between the intervention and the control group (=incremental costs) by the difference in QALYs between the two groups (=incremental QALYs) results in the incremental cost-effectiveness ratio (ICER) calculated as: ICER = (cost_I_– cost_NI_)/(QALY_I_-QALY_NI_), where ‘I’ stands for intervention and ‘NI’ for no intervention.

**Figure 1:**
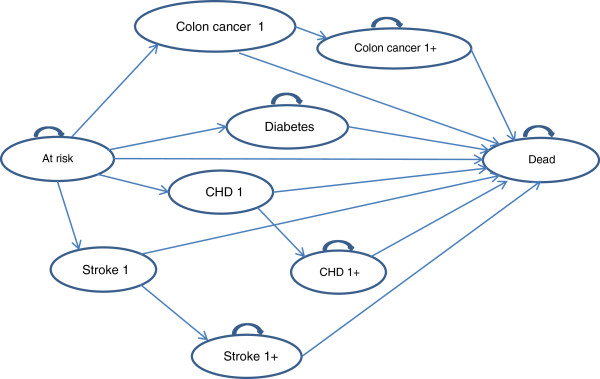
**Markov decision-analytic model.**

The Markov model was based on a published model [17] and further developed using ©Microsoft Excel (Microsoft Corporation, Redmond, WA, US) to account for the specific context and characteristics of the current study. Nine possible states were included in the model (Figure 1): (i) population at risk (‘at risk’), (ii) type 2 diabetes (‘diabetes’), (iii) CHD, first year (‘CHD 1’), (iv) CHD, following years (‘CHD 1+’), (v) stroke, first year (‘stroke 1’), (vi) stroke, following years (‘stroke 1+’), (vii) colon cancer, first year (‘colon cancer 1’), (viii) colon cancer, following years (‘colon cancer 1+’), and (ix) dead (‘dead’). The time horizon of the model was 20 years including 20 one-year periods (called ‘cycles’).

All individuals start in the ‘at risk’ state. During each cycle, an individual has a risk to move to one of the disease states or to ‘dead’. Once an individual is suffering from diabetes, he or she can only remain in that state or move to the ‘dead’ state. Patients suffering from stroke move to the ‘stroke 1’ state. Once a patient has had a stroke, he or she can only move to the ‘stroke 1+’ state or to ‘dead’. Patients who have had a fatal stroke move to the ‘dead’ state after being in the ‘stroke 1’ state for one cycle. Patients in the ‘stroke 1+’ state can only stay in that state or move to ‘dead’. Transitions throughout the model for patients suffering from CHD or colon cancer are analogous as for stroke. CHD includes myocardial infarction and stable or unstable angina. Once a patient enters the ‘dead’ state, no further transitions are possible, as this is the final state.

### Clinical data inputs: disease and mortality transition probabilities

First, the risk of developing diabetes [18, 19], stroke [19, 20], CHD [18, 19], and colon cancer [21] for the general population was calculated. Second, the calculated transition probabilities were multiplied with a relative risk (RR) factor as it is known that individuals with MD are at a greater risk of having diabetes (RR 1.77) [22], stroke (RR 1.77) [23], CHD (RR 18-49 years: 1.42; RR 50-75 years: 1.01) [23], and colon cancer (RR 2.90) [24]. The transition probability from diabetes to colon cancer was calculated by multiplying the transition from ‘at risk’ to diabetes with a RR of 1.33 [25], since diabetic patients have a greater risk of developing colon cancer compared to those without diabetes. The mortality probabilities were obtained from the literature or they were calculated by multiplying national mortality probabilities [26] with the RR of dying from one of the diseases included in the model. Ubink-Veltmaat et al. [27] found a 40% mortality increase in people with diabetes compared to the general Dutch population. A twofold mortality risk was found among stroke patients compared to the Flemish general population [20]. Age- and sex-specific CHD mortality was derived from a study on CHD-mortality in the Netherlands [28]. Colon cancer mortality was obtained from the ‘Cancer survival in Belgium 2004-2008’ report [29]. An overview of the transition probabilities used in the model can be found in Table 1.

**Table 1:** **Age- and gender- dependent probabilities (%) for developing diabetes, stroke, coronary heart disease or colon cancer and associated mortality (%)**

	Diabetes				Coronary heart disease						Stroke						Colon cancer				Overall mortality	
					Incidence		Case fatality rate				Incidence		Case fatality rate									
	Incidence		Case fatality rate				1st year		Follow up				1st year		Follow up		Incidence		Case fatality rate			
Age (years)	M	W	M	W	M	W	M	W	M	W	M	W	M	W	M	W	M	W	M	W	M	W
20-24	0.06	0.05	0.12	0.04	0.01	0.01	5.50	7.30	4.87	7.74	0.02	0.02	13.00	25.00	0.16	0.06	0.01	0.01	6.62	5.67	0.08	0.03
25-29	0.10	0.08	0.13	0.06	0.02	0.02	5.50	7.30	4.87	7.74	0.03	0.03	13.00	25.00	0.18	0.08	0.01	0.01	6.62	5.67	0.09	0.04
30-34	0.17	0.14	0.13	0.06	0.05	0.03	5.50	7.30	4.87	7.74	0.05	0.04	13.00	25.00	0.18	0.09	0.01	0.01	6.62	5.67	0.09	0.04
35-39	0.28	0.23	0.19	0.11	0.10	0.05	5.50	7.30	4.87	7.74	0.07	0.06	13.00	25.00	0.26	0.15	0.03	0.03	6.62	5.67	0.12	0.07
40-44	0.48	0.39	0.26	0.16	0.19	0.08	5.50	7.30	4.87	7.74	0.11	0.09	13.00	25.00	0.36	0.23	0.04	0.04	6.62	5.67	0.15	0.10
45-49	0.78	0.63	0.44	0.27	0.35	0.13	5.50	7.30	4.87	7.74	0.16	0.13	13.00	25.00	0.61	0.38	0.09	0.09	6.62	5.67	0.24	0.16
50-55	1.18	0.96	0.74	0.44	0.41	0.16	5.50	7.30	4.87	7.74	0.25	0.20	36.00	18.00	1.03	0.63	0.16	0.16	7.70	6.89	0.39	0.27
56-59	1.65	1.34	1.19	0.64	0.63	0.27	15.30	17.90	13.56	18.97	0.37	0.30	36.00	18.00	1.65	0.90	0.26	0.26	7.70	6.89	0.61	0.37
60-64	2.08	1.69	1.82	0.98	0.89	0.41	15.30	17.90	13.56	18.97	0.55	0.44	24.00	23.00	2.53	1.39	0.37	0.38	7.70	6.89	0.90	0.54
65-69	2.46	2.00	2.58	1.36	1.17	0.62	15.30	17.90	13.56	18.97	0.83	0.67	24.00	23.00	3.59	1.93	0.60	0.60	9.77	8.91	1.28	0.74

### Effect of the health promotion intervention

A simulation of the evolution of the cohort was made based on the change in Body Mass Index (BMI) between the intervention group and the control group and the age- and gender-dependent associated risk of developing BMI-related diseases. The effectiveness study [15] showed a small but significant difference in BMI change of 0.20 kg/m^2^ between the two study groups. The results of previous studies suggested that a reduction of one BMI unit results in a decreased risk of developing diabetes (men: 13.0%, women: 11.0%) [30], CHD (men: 4.7%, women: 5.7%) [31], stroke (men: 6.0%, women: 8.5%) [32, 33], and colon cancer (men: 5.2%, women: 2.0%) [34]. The risk reductions associated with BMI decrease applied in the Markov model were subsequently calculated using the data derived from these studies (Table 2).

**Table 2:** **Input parameters for the Markov decision-analytic model - base case**

Parameter	Base case	SE	Distribution	Reference
**RR reduction (%)**				
Diabetes - men	2.60	0.003	lognormal	[30]
Diabetes - women	2.20	0.002	lognormal	[30]
CHD - men	0.94	0.001	lognormal	[31]
CHD - women	1.14	0.001	lognormal	[31]
Stroke - men	1.20	0.001	lognormal	[32]
Stroke - women	1.70	0.002	lognormal	[33]
Colon cancer - men	1.04	0.001	lognormal	[34]
Colon cancer - women	0.40	0.001	lognormal	[34]
**Cost/year data input (€)** ^**1**^				
Diabetes	3,312	331	gamma	[35]
CHD first year	4,386	439	gamma	[36–38]
CHD subsequent years	1,183	118	gamma	[36–38]
Stroke first year	13,319	1,332	gamma	[36]
Stroke subsequent years	4,756	476	gamma	[36]
Colon cancer	9,575	958	gamma	[36]
Intervention cost^2^	35	3.50	gamma	[15]
Intervention cost^3^	21	2.13	gamma	[15]
**Utilities**				
At risk	0.71	0.04	beta	[40]
Diabetes	0.63	0.06	beta	[17]
CHD 1	0.47	0.05	beta	[17]
CHD 1+	0.56	0.06	beta	[17]
Stroke 1	0.50	0.05	beta	[17]
Stroke 1+	0.50	0.05	beta	[17]
Colon cancer 1	0.64	0.06	beta	[17]
Colon cancer 1+	0.64	0.06	beta	[17]
Dead	0			

RR, relative risk; CHD, coronary heart disease.

^1^expressed in the year 2011 euros; ^2^intervention cost for the years 1,6,11,16.

^3^intervention cost for the other years.

### Cost data input: disease costs and intervention costs

A public payer perspective was considered including only direct medical costs (Table 2). The costs are expressed in the year 2011 euros. The total diabetes cost was calculated accounting for the proportion of diabetic patients with no complications, micro vascular and/or macro vascular complications [35]. For stroke and CHD, first year costs and following year costs were taken separately into account as the cost related to a newly diagnosed cardiovascular disease was found to be different from the cost for those already suffering longer from stroke or CHD [36–38]. The colon cancer cost was obtained from a health economic evaluation of exercise in the prevention of cardiovascular and other prosperity diseases [36]. Future costs were discounted at 3% [39]. The intervention cost/patient included the use of a pedometer (13.70€), staff cost (20.57€), administrative costs (0.48€), and the intervention manual cost (0.25€). The staff cost/participant was calculated taking into account the total intervention duration time, the number of participating SHOs and individuals, and the time investment (hourly wages). An effective duration of five years was considered for the pedometers, so this cost was taken into account in the Markov model every five years.

### Health-related quality of life (utilities)

The ‘at risk’ utility, i.e. the utility for patients without a history of one of the diseases included in the model was set at 0.71 [40]. The disease-specific utilities were derived from a health economic evaluation of a community-based PA intervention [17] (Table 2). QALYs were calculated by multiplying the utilities with the number of life years an individual is living with one of the diseases included in the model. Future QALYs were discounted at 1.5% [39].

### Scenario analysis and sensitivity analyses

Health economic evaluations are frequently characterized by some degree of uncertainty or methodological considerations [41]. In the current study, scenario analyses and sensitivity analyses were performed to tackle this uncertainty. In the scenario analysis, four alternative modelling assumptions were assessed. First, full compliance with the intervention was assumed. For this analysis, a mean change of 0.33 kg/m^2^ (i.e. the mean BMI change of the participants who completed the programme) [15] was considered. In a second scenario, the effects on the costs and the QALYs of offering the programme twice a year maintaining a mean BMI change of 0.20 kg/m^2^ was analysed. In the base case analysis, no increase in HRQOL as a result of the BMI decrease was accounted for. So, in a third scenario, in the intervention group, a utility gain of 0.021 per unit BMI decrease was assumed based on the findings of a study of valuing HRQOL in diabetes patients [42]. In that study, a utility loss of 0.021 per unit BMI increase was accounted for. So, we assumed a utility gain of 0.021 per unit BMI decrease. In the base case analysis, a 20-year time horizon was used maintaining the same intervention effect. As a fourth scenario analysis, a more conservative analysis was conducted considering a five-year time horizon. One-way sensitivity analyses made it possible to assess the effects of key input parameters (intervention cost and effect, disease costs, and RR reductions of the diseases associated with a BMI decrease) on the ICER, by varying them separately. A probabilistic sensitivity analysis was performed to assess the uncertainty for the key input parameters by varying them concurrently. Cost data were assumed to follow a gamma distribution, utilities follow a beta distribution and risk reductions a lognormal distribution [43].

### Ethics

The study was in compliance with the Helsinki Declaration and permission to perform the study was obtained from the Ethics Committee of the University Hospital of Ghent.

## Results

### Base case analysis

For the treatment as usual group, the average QALYs amounted to 11.59 and 12.04 with a cost of 8,352€ and 7,688€ for men and women respectively. The implementation of the health promotion intervention resulted in a limited QALY gain of 0.01 in both men and women. The total discounted costs in the intervention group were 8,579€ in men and 7,951€ in women, resulting in a delta cost between intervention and no intervention of 228€ and 263€ in men and women respectively. This resulted in an ICER of 27,096€/QALY in men and 40,139€/QALY in women (Table 3).

**Table 3:** **Cost-effectiveness results in men and women (base case and scenario analysis)**

	Control		Intervention		∆ QALY	∆ Cost (€)	ICER (€/QALY)
	QALYs	Cost (€)	QALYs	Cost (€)			
**Men**							
**Base case**	11.59	8,352	11.60	8,579	0.01	228	27,096
**Scenario 1**	11.59	8,352	11.61	8,494	0.01	142	10,241
**Scenario 2**	11.59	8,352	11.60	8,938	0.01	586	69,754
**Scenario 3**	11.59	8,352	11.66	8,579	0.07	228	3,357
**Scenario 4**	4.05	980	4.05	1,095	0.00	115	190,647
**Women**							
**Base case**	12.04	7,688	12.05	7,951	0.01	263	40,139
**Scenario 1**	12.04	7,688	12.05	7,881	0.01	193	17,857
**Scenario 2**	12.04	7,688	12.05	8,320	0.01	632	96,567
**Scenario 3**	12.04	7,688	12.11	7,951	0.07	263	3,766
**Scenario 4**	4.08	807	4.08	926	0.00	119	266,700

Scenario 1, full compliance with the programme; scenario 2, offering the programme twice a year.

Scenario 3, increase in quality of life as a result of the BMI-decrease in the intervention group.

Scenario 4: time horizon of 5 years.

ICER, Incremental Cost-Effectiveness Ratio; QALY, Quality Adjusted Life Year.

### Scenario analysis of alternative modelling assumptions

Applying the scenario of full compliance with the programme resulted in an ICER of 10,241€/QALY in men and 17,857€/QALY in women. An increase in HRQOL as a result of the BMI decrease resulted in an ICER of 3,357€/QALY in men and 3,766€/QALY in women. Worse results were found in the scenario offering the programme twice a year (Table 3). Taking into account a five-year time horizon resulted in an ICER of 190,647€/QALY in men and 266,700€/QALY in women.

### Sensitivity analyses

**Figure 2:**
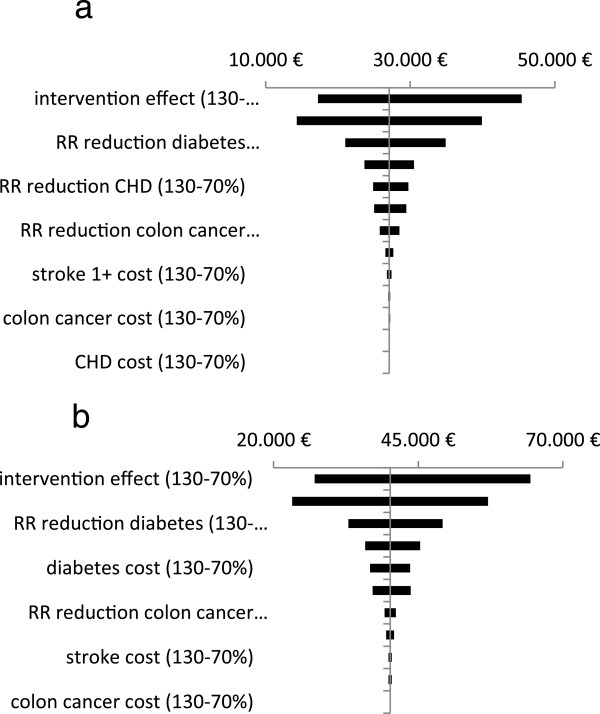
**One-way sensitivity analysis: effects on cost/QALY. (a)** Men. **(b)** Women.

**Figure 3:**
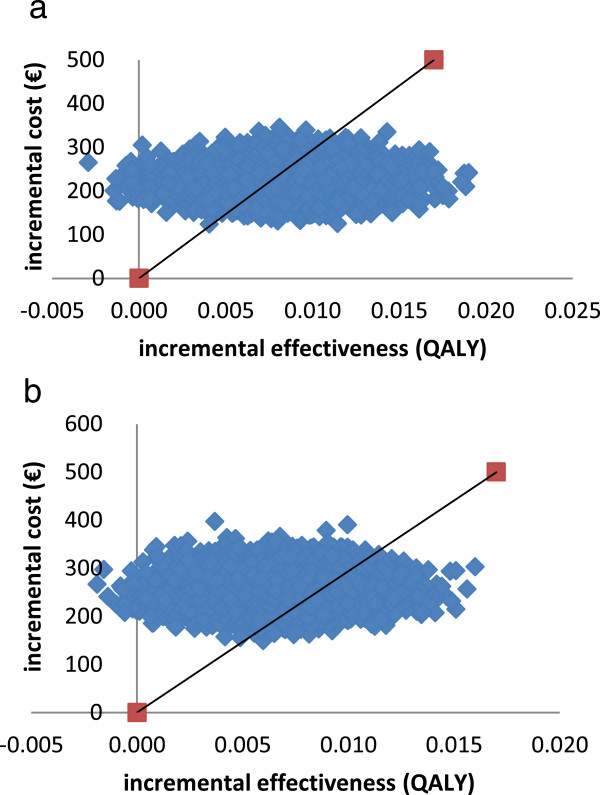
**Probabilistic sensitivity analysis. (a)** Men. **(b)** Women.

The results of the one-way sensitivity analyses are shown using Tornado diagrams (Figure 2a and b). From this figure it can be concluded that the model is most sensitive to the intervention effect and to the intervention cost in both men and women. Varying other input parameters had less influence on the results. The findings of the probabilistic sensitivity analysis are shown in cost-effectiveness planes (Figure 3a and b). The points to the right of the threshold line refer to a cost-effectiveness ratio less than 30,000€/QALY. Based on 10,000 simulations, 95% credible intervals (CI) could be generated. The health promotion programme resulted in an average QALY gain of 0.008 (95% CI 0.003-0.014) at an average cost of 221€ (95% CI 168€-278€) in men and in an average QALY gain of 0.007 (95% CI 0.002-0.011) at an average cost of 256€ (95% CI 201€-316€) in women. In men, an average ICER of 26,336€/QALY (95% CI 14,439-83,209€/QALY) was found, while in women the ICER was 39,094€/QALY (95% CI 21,573-120,541€/QALY).

## Discussion

The aim of the study was to evaluate the cost-effectiveness of a health promotion programme targeting PA and healthy eating in individuals with MD living in sheltered housing in the Flanders region (Belgium). A Markov decision-analytic model providing information on the costs and on the health effects (expressed as QALYs) related to the programme was used. The base case analysis showed an ICER of 27,096€/QALY in men and 40,139€/QALY in women. Assuming a cost-effectiveness threshold of about 30,000€/QALY in Belgium as recommended by the Belgian Healthcare Knowledge Centre [44], it can be concluded that the intervention was cost-effective in men but not in women. Mixed results were identified from the scenario analyses. The most promising outcome was found when an increase in HRQOL associated with a BMI decrease was assumed. The model was most sensitive to the input parameters ‘intervention effect’ and ‘intervention cost’ as demonstrated with the one-way sensitivity analyses.

In the base case, the health promotion programme was found to be borderline cost-effective in men and not cost-effective in women. The explanation for this result is likely the fact that a limited, although statistically significant, BMI change of 0.20 kg/m^2^, drawn from the effectiveness study [15], was used to calculate the risk reductions for the diseases included in the model. This was confirmed by the one-way sensitivity analysis results, identifying the ‘intervention effect’ as the input parameter most influencing the outcome. The limited change in BMI may be explained by the relatively short intervention duration of ten weeks. A more promising result was found if a BMI change of 0.33 kg/m^2^ assuming full compliance with the programme was considered. It can yet be discussed to what extent full compliance with the programme is achievable in populations with MD. Previous qualitative research identified a number of barriers hampering the participation of psychiatric patients in health promotion interventions [45]. Applying the scenario in which an increase in HRQOL as a result of BMI decrease was assumed, resulted in a conclusive cost-effective outcome in both men and women. This result must however be interpreted cautiously. Research on the effects of weight loss on HRQOL in people with MD is scarce providing no conclusive evidence that weight loss is associated with an increase in HRQOL [14]. A clearly not cost-effective result was observed considering a five-year time horizon. If the health effects are expressed as life years(LY) gained, the intervention resulted in 0.01 LY gained in men (intervention: 16.62 LY, control: 16.61 LY) and in women (intervention: 17.18 LY, control: 17.17 LY) (data not shown).

In the current healthcare environment, there is growing need on health economic evaluations of such programmes because this can assist regulatory bodies and health insurers establishing priorities within cost-constrained healthcare budgets. In mental health care, health economic research predominantly focused on cost-effectiveness analyses of psychopharmacological drugs [46] and mental health promotion and MD prevention [47]. Although the need for health economic evaluations of health promotion programmes targeting PA and healthy eating in mental health care was already addressed [48], such studies are lacking [14]. A uniform 70% to 130% uncertainty was used in the one-way sensitivity analysis, since the main reason to perform this analysis was to gain insight in those parameters most influencing the study outcome and not to assess the implications on the study outcome of uncertainty in the parameters. The full uncertainty around the input parameters was reflected with the probabilistic sensitivity analysis [41].

Some limitations and considerations need to be addressed. First, a 20-year time horizon assuming that the 10-week health promotion programme was repeated every year maintaining the same BMI change was considered. Studies evaluating the long-term effects of health promotion targeting PA and healthy eating in individuals with MD are yet lacking [14]. So, uncertainty exists concerning the long-term effects on BMI of offering such a programme once a year. The time horizon used in the current study was based on the time horizon used in a previous health economic evaluation of a community-based PA intervention [17]. We are aware that the time horizon is excessive on the basis of evidence found following a 10-week intervention the benefits of which disappeared at six months [15]. There is only limited evidence suggesting that health promotion targeting PA and healthy eating can result in longer-term weight loss. Unick et al. [49] found that weight loss was possible in overweight and obese diabetes patients following a repeated health promotion intervention targeting PA and healthy eating during a 4-year time period. Nevertheless, there is no evidence supporting that a repeated health promotion programmme would maintain the same effect during a 20-year time period. So, as a more conservative approach, an additional analysis was performed considering a five-year time period resulting in a clearly not cost-effective outcome in both men and women. On the other hand, the use of extensive time horizons in health economic evaluations is quite common [17, 36, 50]. Second, the model is a simplification of real life because model complexity prevented us to for example allow some combinations of disease states. Efforts to approximate the reality were yet applied, such as the inclusion of a transition probability from diabetes to colon cancer. No transitions from diabetes to CHD and stroke were possible, but the proportion of macro vascular complications such as CHD and stroke [51] was accounted for in the calculation of the diabetes cost. Costs related to follow-up after being diagnosed with one of the diseases included in the model were accounted for in the follow-up states (the states ‘1+’ in the Markov model). Third, the disease transition and mortality probabilities were retrieved from the literature and nationally available data. For some probabilities, national data were absent, so data from other countries was used. This may have resulted in an underestimation or overestimation of some probabilities included in the model. A utility of 0.71 [40] found in a sample of schizophrenia and major depressive disorder patients was used for the ‘at risk’ state. We are aware that utilities may differ according to the psychiatric diagnosis [52]. Nevertheless, the use of the 0.71 utility in our study is likely to be a reasonable reflection of the reality as about two-thirds of our study population consisted of schizophrenia and mood disorder patients. It is also important to note that utility weights may vary according to the measurement instrument being used. Lamers et al. [53] compared the use of the EQ-5D and the SF-6D questionnaires in a MD patients sample. It was found that the use of the EQ-5D resulted in larger health gains and consequent lower cost-utility ratios compared with the SF-6D. In one of the scenario analyses, an increase in HRQOL due to a BMI decrease was assumed. It can be argued that there is a risk of double counting of the utility benefit since the risk reduction of disease already incorporates a gain in QALYs. In the scenario analysis, the utility increase was only accounted for in the intervention group in the ‘at risk’ state and not in the different disease states.

## Conclusions

In conclusion, as far as is known to the authors, this is the first study assessing the cost-effectiveness of a health promotion intervention targeting PA and healthy eating in individuals with MD. It was found that the health promotion intervention is likely to be cost-effective on the long-term if the programme would be repeated every year maintaining the same effect. This rather optimistic finding must be cautiously interpreted since there is no evidence supporting the long-term effectiveness of such interventions on BMI. Further research examining the cost-effectiveness of health promotion interventions in populations with MD is required. Further health economic evaluations of health promotion programmes is also required accounting for other study conditions such as individually-based programmes, programmes with longer duration and/or delivered in other settings. Such research has a substantial social value because healthcare budgets are limited, hence policy makers are facing the problem how to set priorities in the allocation of healthcare resources to medical or public health interventions.
